# Supplementary material for: Inhibition of phosphatidylinositol 3‐kinase α (PI3Kα) prevents heterotopic ossification
Source: EMBO Mol Med. 2019 Aug 2;11(9):e10567. doi: 10.15252/emmm.201910567 (PMC6728602; doi:10.15252/emmm.201910567)
Supplement: Supplementary file 1 — Appendix [file EMMM-11-e10567-s001.pdf]

Appendix

Table of contents

Appendix Table S1. List of qRT-PCR TaqMan probes.

Appendix Table S2. Detailed p-values.

Gene	Assay ID
Id1	Mm00775963_g1 (ThermoFisher Scientific)
Sox9	Mm00448840_m1 (ThermoFisher Scientific)
Dlx5	Mm01161781_m1 (ThermoFisher Scientific)
Sp7	Mm00504574_m1 (ThermoFisher Scientific)
Col1a1	Mm00801666_g1 (ThermoFisher Scientific)
Runx2	Mm03003491_m1 (ThermoFisher Scientific)
Bglap	Mm03413826_mH (ThermoFisher Scientific)
Acvr1	Mm01331067_m1 (ThermoFisher Scientific)
Tbp	Mm01277042_m1 (ThermoFisher Scientific)

Appendix Table S1. List of qRT-PCR TaqMan probes.

Appendix Table S2			
**Figure**	**Compared groups**	**Symbol**	**p-value**
**Figure 1A**	- vs. WT	***	< 0,0001
	- vs. RH	***	< 0,0001
	- vs. QD	***	< 0,0001
**Figure 2**	**ID1 Control**		
	Control - A66 - Control + A66		
	-	*	0,0139
	**ID1 BMP2**		
	BMP2 - A66 - BMP2 + A66		
	-	***	0,0002
	WT	*	0,0131
	RH	*	0,02
	QD	**	0,0017
	**ID1 BMP6**		
	BMP6 - A66 - BMP6 + A66		
	-	**	0,0058
	WT	**	0,0053
	RH	*	0,024
	QD	***	0,0002
	**ID1 Activin A**		
	Activin A - A66 - Activin A + A66		
	QD	**	0,0042
	Activin A - A66		
	WT vs. RH	##	0,0042
	WT vs. QD	###	< 0,0001
	**Sox9 Control**		
	Control - A66 - Control + A66		
	-	***	< 0,0001
	WT	***	< 0,0001
	RH	**	0,0088
	QD	**	0,0036
	**Sox9 BMP2**		
	BMP2 - A66 - BMP2 + A66		
	WT	*	0,0374
	RH	*	0,0137
	QD	*	0,0258
	**Sox9 BMP6**		
	BMP6 - A66 - BMP6 + A66		
	-	***	0,0002
	WT	***	< 0,0001
	QD	**	0,0036
	**Sox9 Activin A**		
	Activin A - A66 - Activin A + A66		
	-	***	< 0,0001
	WT	***	< 0,0001
	RH	***	< 0,0001
	QD	***	< 0,0001
	Activin A - A66		

WT vs. RH	##	0,0099
**Dlx5 Control**		
Control - A66 - Control + A66		
-	***	0,0002
WT	***	< 0,0001
RH	***	0,0003
QD	***	< 0,0001
Control - A66		
WT vs. QD	###	< 0,0001
**Dlx5 BMP2**		
BMP2 - A66 - BMP2 + A66		
-	*	0,0341
WT	**	0,0077
RH	***	< 0,0001
QD	***	< 0,0001
BMP2 - A66		
WT vs. RH	###	< 0,0001
WT vs. QD	###	< 0,0001
**Dlx5 BMP6**		
BMP6 - A66 - BMP6 + A66		
-	***	< 0,0001
WT	***	< 0,0001
RH	**	0,0046
QD	***	< 0,0001
BMP6 - A66		
WT vs. QD	###	< 0,0001
**Dlx5 Activin A**		
Activin A - A66 - Activin A + A66		
-	*	0,0248
RH	***	< 0,0001
QD	***	< 0,0001
Activin A - A66		
WT vs. RH	###	< 0,0001
WT vs. QD	###	< 0,0001
**Sp7 Control**		
Control - A66 - Control + A66		
RH	**	0,0058
QD	**	0,0021
Control - A66		
WT vs. RH	##	0,0058
WT vs. QD	###	0,0003
**Sp7 BMP2**		
BMP2 - A66 - BMP2 + A66		
QD	***	0,0005
BMP2 - A66		
WT vs. RH	##	0,001
WT vs. QD	###	< 0,0001
**Sp7 BMP6**		
BMP6 - A66 - BMP6 + A66		
QD	***	0,0008

	BMP6 - A66		
	WT vs. QD	###	< 0,0001
	**Sp7 Activin A**		
	Activin A - A66 - Activin A + A66		
	RH	***	< 0,0001
	QD	**	0,0029
	Activin A - A66		
	WT vs. RH	###	< 0,0001
	WT vs. QD	###	0,0001
**Figure 3D**	ACVR1 Q207D DMSO vs. ACVR1 Q207D Intermittent BYL719	***	0,0007
	ACVR1 Q207D DMSO vs. ACVR1 Q207D daily BYL719	**	0,0074
**Figure 5**			
**5A**	**16**		
	ACVR1 QD DMSO vs. ACVR1 QD Daily BYL719	#	0,0127
	**18**		
	ACVR1 QD DMSO vs. ACVR1 QD Daily BYL719	##	0,0094
	**20**		
	ACVR1 QD DMSO vs. ACVR1 QD Daily BYL719	##	0,0025
	**22**		
	ACVR1 QD DMSO vs. ACVR1 QD Daily BYL719	###	< 0,0001
	**24**		
	ACVR1 QD DMSO vs. ACVR1 QD Daily BYL719	###	< 0,0001
	**26**		
	Control DMSO vs. Control Daily BYL719	**	0,004
	ACVR1 QD DMSO vs. ACVR1 QD Daily BYL719	###	< 0,0001
	**28**		
	Control DMSO vs. Control Daily BYL719	***	0,0001
	ACVR1 QD DMSO vs. ACVR1 QD Daily BYL719	###	< 0,0001
	**30**		
	Control DMSO vs. Control Daily BYL719	***	< 0,0001
	ACVR1 QD DMSO vs. ACVR1 QD Daily BYL719	###	< 0,0001
**5B**	CONTROL:DMSO vs. CONTROL:Daily BYL719	***	0,0003
	CONTROL:Intermittent BYL719 vs. CONTROL:Daily BYL719	**	0,0031
	ACVR1 Q207D:DMSO vs. ACVR1 Q207D:Daily BYL719	***	< 0,0001
	ACVR1 Q207D:Intermittent BYL719 vs. ACVR1 Q207D:Daily BYL719	***	< 0,0001
**5C**	**Cortical Bone**		
	**Bone Volume**		
	CONTROL:DMSO vs. CONTROL:Daily BYL719	***	< 0,0001
	CONTROL:Intermittent BYL719 vs. CONTROL:Daily BYL719	***	< 0,0001
	ACVR1 Q207D:DMSO vs. ACVR1 Q207D:Daily BYL719	***	< 0,0001
	ACVR1 Q207D:Intermittent BYL719 vs. ACVR1 Q207D:Daily BYL719	***	< 0,0001
	**Bone Perimeter**		
	ACVR1 Q207D:DMSO vs. ACVR1 Q207D:Daily BYL719	***	< 0,0001
	ACVR1 Q207D:Intermittent BYL719 vs. ACVR1 Q207D:Daily BYL719	***	< 0,0001
	**Cortical Thickness**		
	CONTROL:DMSO vs. CONTROL:Daily BYL719	***	< 0,0001

	CONTROL:Intermittent BYL719 vs. CONTROL:Daily BYL719	***	< 0,0001
	ACVR1 Q207D:DMSO vs. ACVR1 Q207D:Daily BYL719	***	< 0,0001
	ACVR1 Q207D:Intermittent BYL719 vs. ACVR1 Q207D:Daily BYL719	***	< 0,0001
	**Trabecular Bone**		
	**Bone Volume Fraction**		
	ACVR1 Q207D:DMSO vs. ACVR1 Q207D:Daily BYL719	***	< 0,0001
	ACVR1 Q207D:Intermittent BYL719 vs. ACVR1 Q207D:Daily BYL719	***	< 0,0001
	**Trabecular Number**		
	ACVR1 Q207D:DMSO vs. ACVR1 Q207D:Daily BYL719	***	< 0,0001
	ACVR1 Q207D:Intermittent BYL719 vs. ACVR1 Q207D:Daily BYL719	***	< 0,0001
	**Trabecular Thickness**		
	ACVR1 Q207D:DMSO vs. ACVR1 Q207D:Daily BYL719	***	< 0,0001
	ACVR1 Q207D:Intermittent BYL719 vs. ACVR1 Q207D:Daily BYL719	***	< 0,0001
**Figure EV1C**	**Dlx5**		
	- A66 vs. 0.5 µm A66	***	0,0004
	- A66 vs. 1 µm A66	***	< 0,0001
	0.5 µm A66 vs. 1 µm A66	*	0,0348
	**Sp7**		
	- A66 vs. 1 µm A66	**	0,0028
	0.5 µm A66 vs. 1 µm A66	*	0,0135
	**Col1a1**		
	- A66 vs. 1 µm A66	**	0,0051
	**Bglap**		
	- A66 vs. 1 µm A66	**	0,0027
**Figure EV2**	**Bglap Control**		
	-:- vs. -:QD	#	0,0231
	-:QD vs. +:QD	*	0,0249
	**Bglap BMP2**		
	-:- vs. -:QD	#	0,0236
	-:QD vs. +:QD	**	0,0072
	**Bglap BMP6**		
	-:QD vs. +:QD	*	0,0259
	**Col1a1 Control**		
	-:- vs. +:-	*	0,0189
	-:WT vs. +:WT	*	0,0123
	-:RH vs. +:RH	***	0,0005
	-:QD vs. +:QD	**	0,003
	**Col1a1 BMP2**		
	-:WT vs. +:WT	*	0,0174
	-:RH vs. +:RH	**	0,0043
	**Col1a1 BMP6**		
	-:QD vs. +:QD	*	0,0495
	**Col1a1 Activin A**		
	-:RH vs. +:RH	*	0,0327
	-:QD vs. +:QD	*	0,0237
**Figure EV3C**	**Id1**		

	- BYL719:RH vs. + BYL719:RH	**	0,0013
	- BYL719:QD vs. + BYL719:QD	*	0,0425
	**Col1a1**		
	- BYL719:- vs. + BYL719:-	***	< 0,0001
	- BYL719:WT vs. + BYL719:WT	***	0,0002
	- BYL719:RH vs. + BYL719:RH	***	< 0,0001
	- BYL719:QD vs. + BYL719:QD	***	< 0,0001
	**Bglap**		
	- BYL719:- vs. + BYL719:-	*	0,0103
	- BYL719:WT vs. + BYL719:WT	**	0,0012
	- BYL719:RH vs. + BYL719:RH	***	< 0,0001
	- BYL719:QD vs. + BYL719:QD	***	0,0001
	**Runx2**		
	- BYL719:RH vs. + BYL719:RH	**	0,0089
	- BYL719:QD vs. + BYL719:QD	*	0,0346
	**Dlx5**		
	- BYL719:- vs. - BYL719:QD	#	0,026
	- BYL719:RH vs. - BYL719:QD	#	0,0467
	- BYL719:QD vs. + BYL719:QD	**	0,0081
	**Sp7**		
	- BYL719:- vs. - BYL719:RH	##	0,0047
	- BYL719:- vs. - BYL719:QD	###	< 0,0001
	- BYL719:WT vs. - BYL719:RH	##	0,0066
	- BYL719:WT vs. - BYL719:QD	###	< 0,0001
	- BYL719:RH vs. - BYL719:QD	##	0,0091
	- BYL719:RH vs. + BYL719:RH	*	0,0239
	- BYL719:QD vs. + BYL719:QD	***	< 0,0001
