# Supplementary material for: Inhibition of phosphatidylinositol 3‐kinase α (PI3Kα) prevents heterotopic ossification
Source: EMBO Mol Med. 2019 Aug 2;11(9):e10567. doi: 10.15252/emmm.201910567 (PMC6728602; doi:10.15252/emmm.201910567)
Supplement: Supplementary file 2 — Expanded View Figures PDF [file EMMM-11-e10567-s002.pdf]

Expanded View Figures

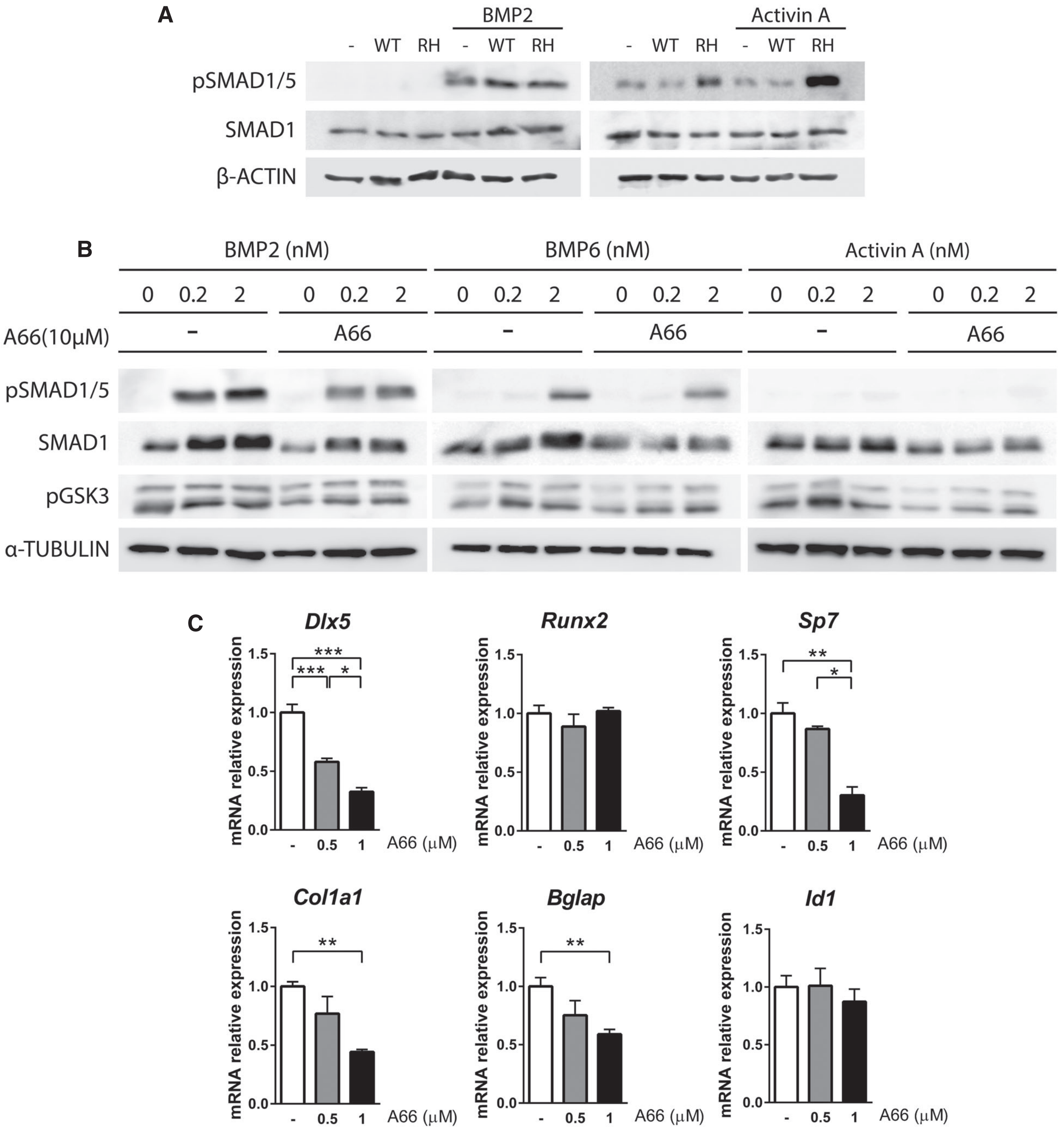

Figure EV1. Dose selection and MSC osteogenic differentiation.

A Immunoblots of MSCs expressing wild-type *Acvr1* (WT) or *Acvr1*[R206H] (RH). MSCs were serum-starved for 16 h and then treated with 2 nM BMP2 or 2 nM activin A for 1 h.

B Immunoblots of MSCs. Cells were serum-starved and treated with 10 μM A66 for 16 h and then treated with the indicated concentrations of BMP2, BMP6, or activin A for 1 h.

C MSCs were cultured for 14 days in osteogenic media in the presence of the indicated concentrations of A66. Data shown as mean ± SEM (n = 5 per group). $^{*}P < 0.05$, $^{**}P < 0.01$, $^{***}P < 0.001$; one-way ANOVA.

Source data are available online for this figure.

© 2019 The Authors

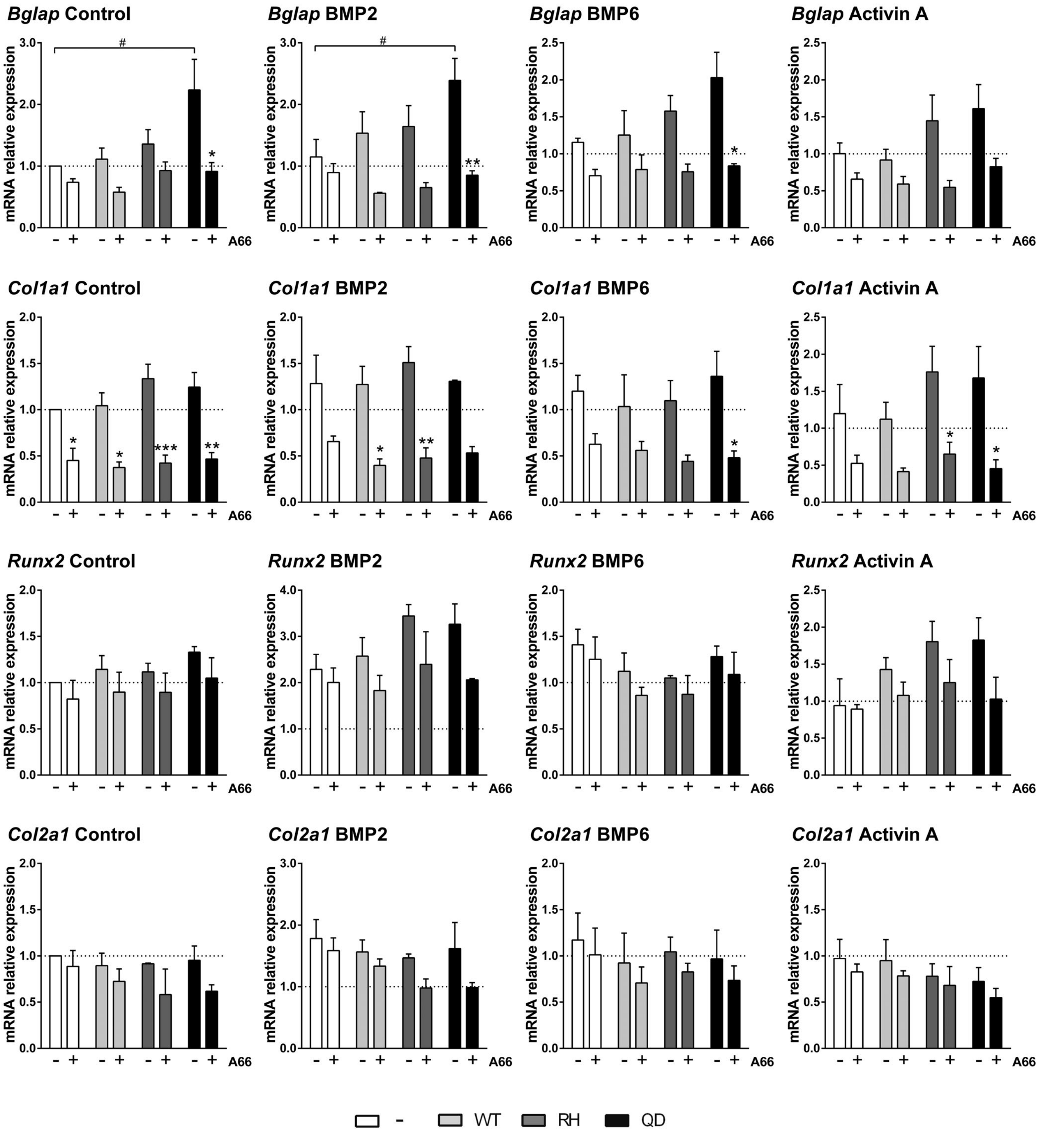

Figure EV2. PI3Kα inhibition reduces the specification of cell progenitors into chondrogenic and osteogenic lineages.

mRNA expression of osteoblast and chondroblast-specific genes of MSCs expressing wild-type *Acvr1*, *Acvr1*^{R206H}, or *Acvr1*^{Q207D}. MSCs were serum-starved and treated with 10 μM A66 for 16 h and then treated with 2 nM BMP2, 2 nM BMP6, or 2 nM activin A for 2 h. Data shown as mean ± SEM (n = 4 per group). * or $^{\#}P < 0.05$, $^{**}P < 0.01$, $^{***}P < 0.001$; two-way ANOVA. Asterisks refer to significance between MSCs treated with or without A66 in each case. Similarly, # refers to significance between *Acvr1*^{R206H} or *Acvr1*^{Q207D} MSCs of each group compared to mock-transfected cells untreated with A66.

Figure EV3. PI3Kα inhibition with BYL719 in MSCs.

A Immunoblots of MSCs. Cells were serum-starved and treated with a range of BYL719 concentrations for 16 h and then with 2 nM BMP2 for 1 h.

B Immunoblots of MSCs expressing wild-type *Acvr1* (WT) or *Acvr1*^{R206H} (RH) variants. MSCs were serum-starved and treated with 10 μM BYL719 for 16 h and then with 2 nM BMP6 or 2 nM activin A for 1 h.

C mRNA expression of osteoblast and chondroblast-specific genes of MSCs expressing wild-type *Acvr1*, *Acvr1*^{R206H}, or *Acvr1*^{Q207D}. MSCs were serum-starved and treated with 10 μM BYL719 for 16 h. Data shown as mean ± SEM (n = 4 per group). * or $^{\#}P < 0.05$, ** or $^{\#\#}P < 0.01$, *** or $^{\#\#\#}P < 0.001$; two-way ANOVA. Asterisks refer to significance between MSCs treated with or without BYL719 in each case. Similarly, # refers to significance between untreated groups transfected with different forms of *Acvr1*.

Source data are available online for this figure.

 © 2019 The Authors

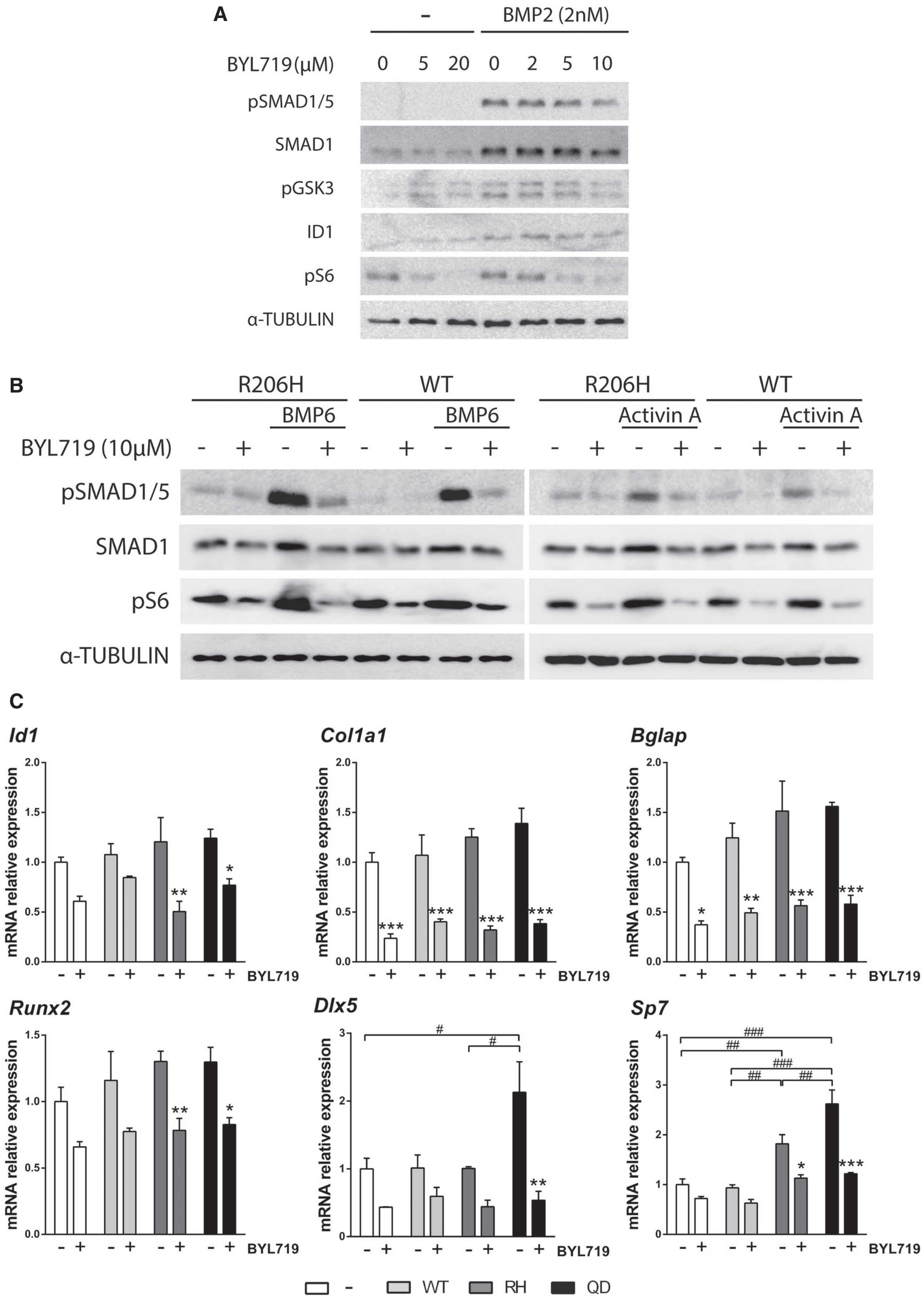
A
-
BMP2 (2nM)
BYL719(μM) 0 5 20 0 2 5 10
pSMAD1/5
SMAD1
pGSK3
ID1
pS6
α-TUBULIN
B
R206H
WT
R206H
WT
BMP6
BMP6
Activin A
Activin A
BYL719 (10μM)
pSMAD1/5
SMAD1
pS6
α-TUBULIN
C
Id1
Col1a1
Bglap
Runx2
Dlx5
Sp7
mRNA relative expression
BYL719
- WT RH QD

Figure EV3.

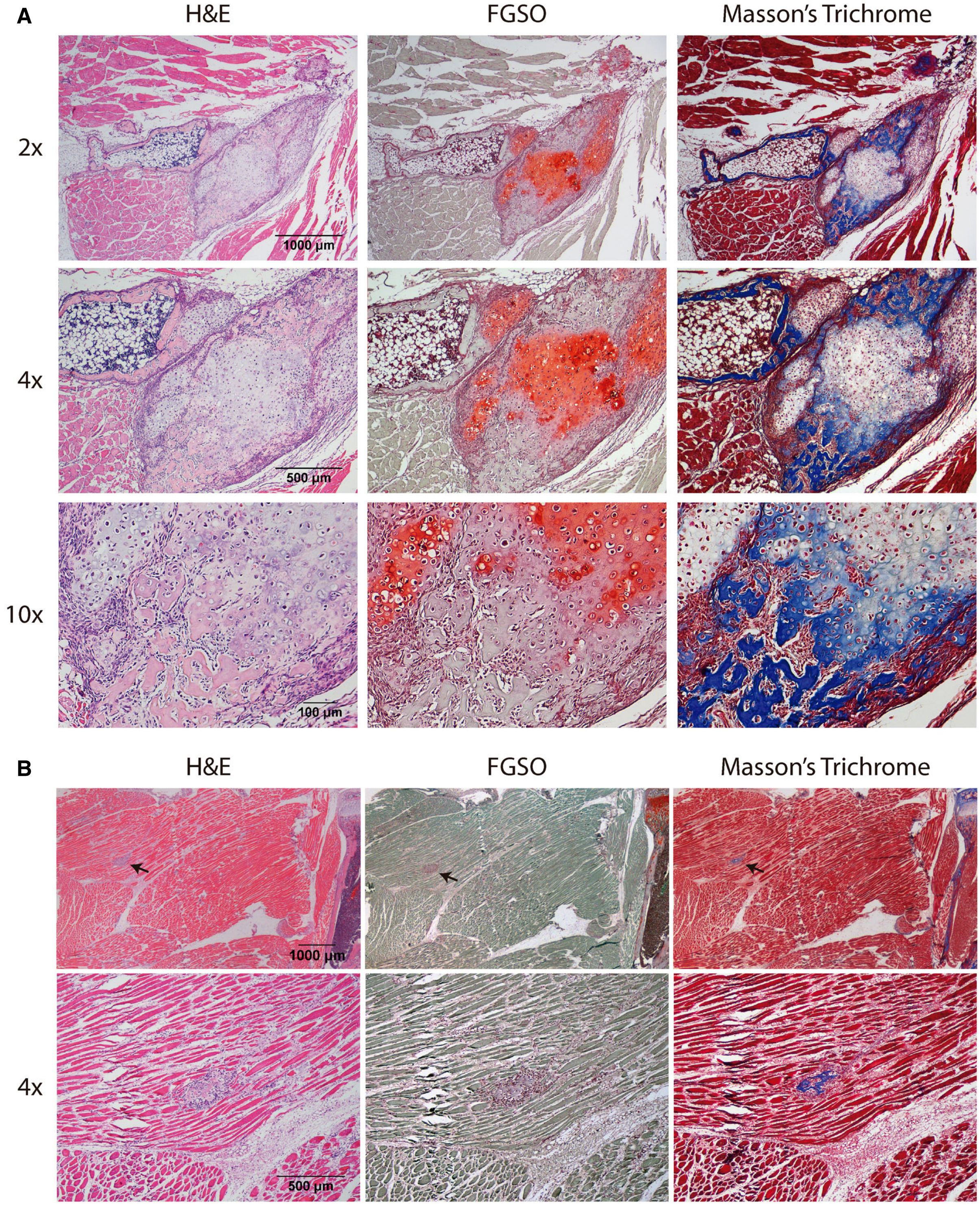

Figure EV4. Detailed histological images of endochondral heterotopic ossification.

A Representative images of HO of *Acvr1*^{Q207D} mice treated with vehicle: hematoxylin and eosin (H&E), fast green/safranin O (FGSO), and Masson's trichrome staining. Microscopy images shown with 2× (scale bar = 1,000 μm), 4× (scale bar = 500 μm), and 10× (scale bar = 100 μm).

B Representative images of the single case of heterotopic ossification in *Acvr1*^{Q207D} mice treated with intermittent BYL719. Images obtained with stereomicroscope (scale bar = 1,000 μm). Microscopy images shown with 4× (scale bar = 500 μm). Black arrows show the localization of heterotopic ossification.

© 2019 The Authors
