# Supplementary figures and images for: Inhibition of phosphatidylinositol 3‐kinase α (PI3Kα) prevents heterotopic ossification
Source: EMBO Mol Med. 2019 Aug 2;11(9):e10567. doi: 10.15252/emmm.201910567 (PMC6728602; doi:10.15252/emmm.201910567)

A

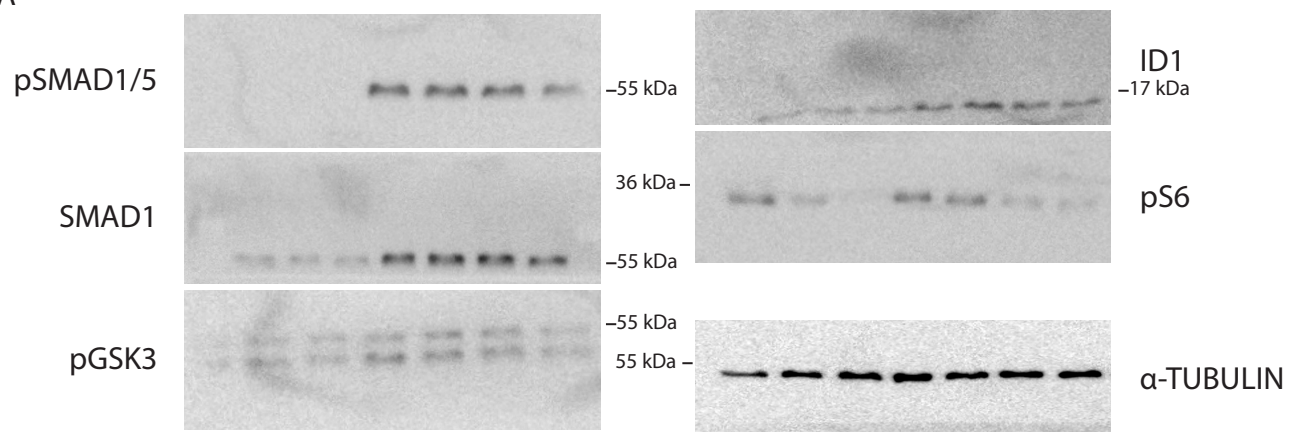

## B

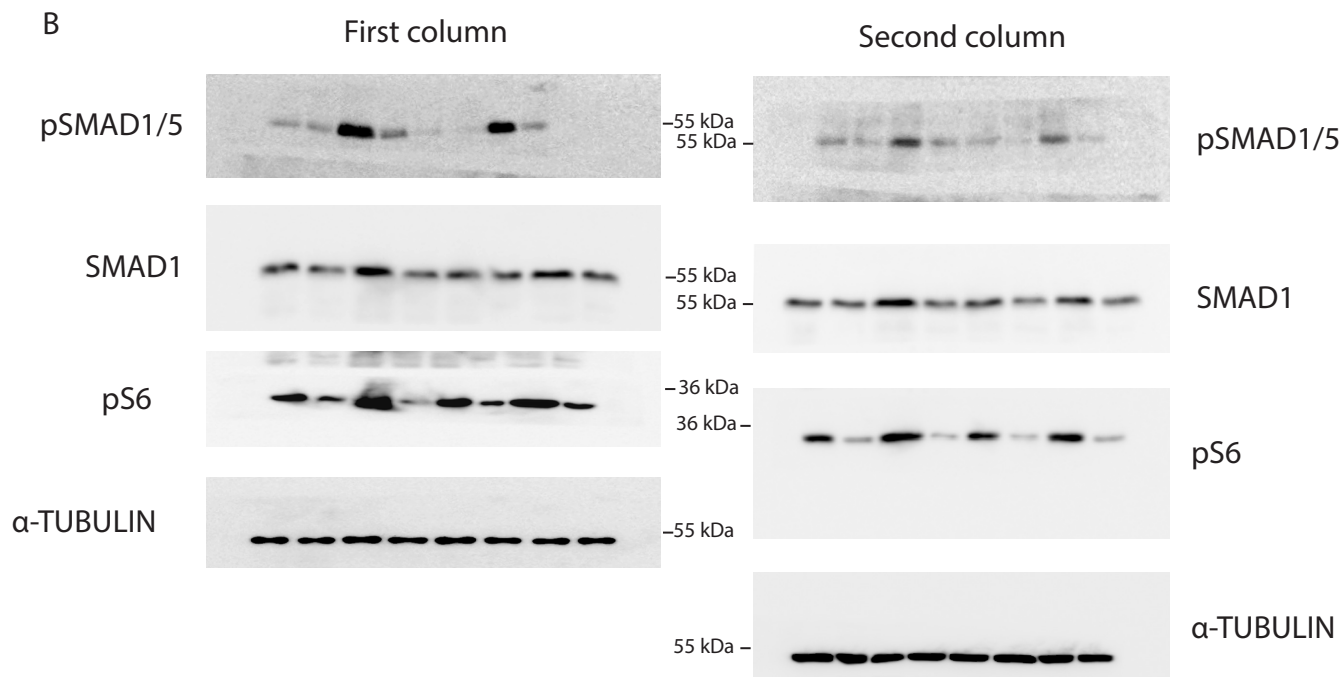

Supplement: Supplementary file 6 — Source Data for Expanded View [file EMMM-11-e10567-s007.zip › EMM-2019-10567_SourceDataForExpandedView/EMM-2019-10567_SourceDataForExpandedView3.pdf]

Source Data Expanded View Figure EV1

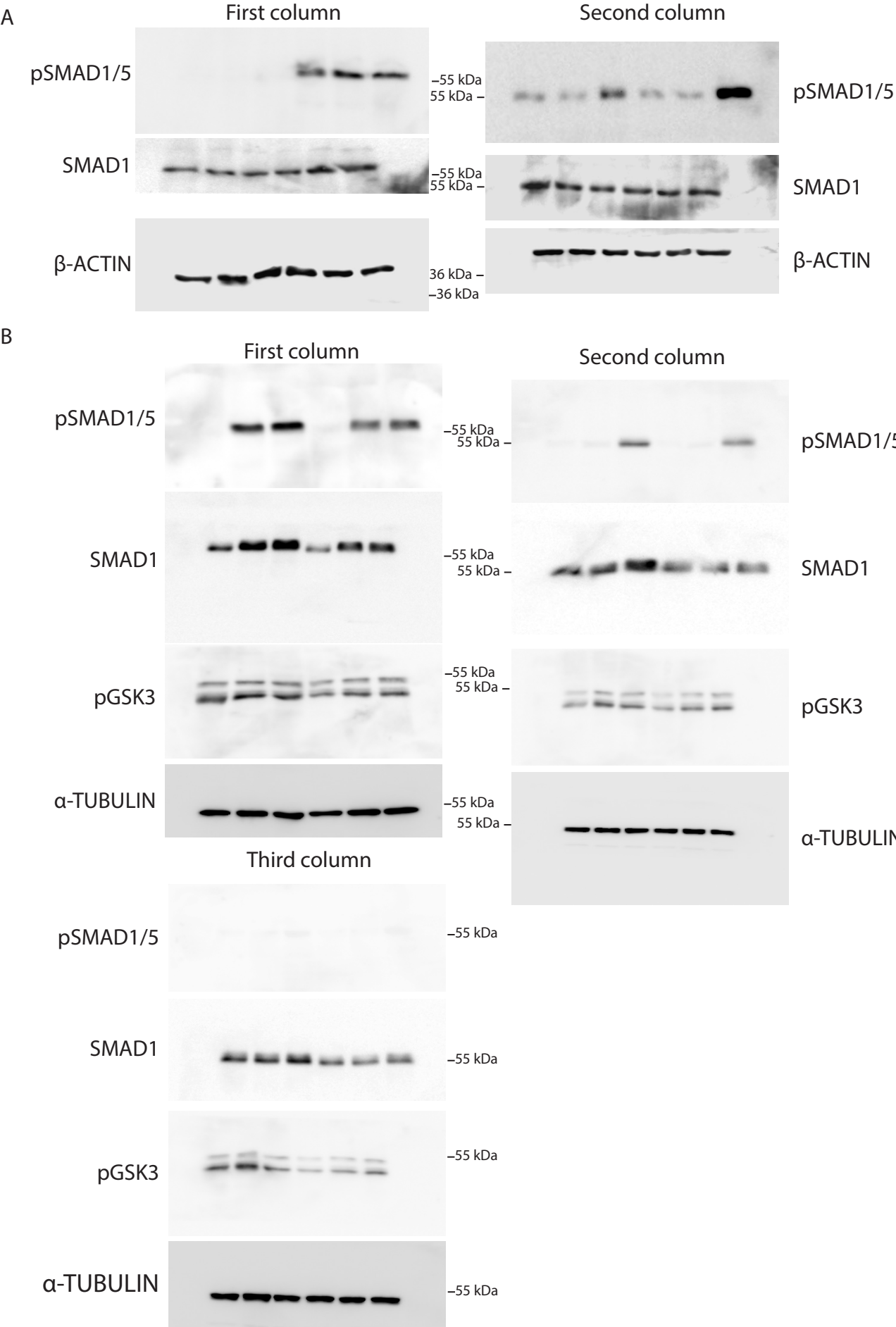

Supplement: Supplementary file 6 — Source Data for Expanded View [file EMMM-11-e10567-s007.zip › EMM-2019-10567_SourceDataForExpandedView/EMM-2019-10567_SourceDataForExpandedView1.pdf]

Source Data Figure 1

B

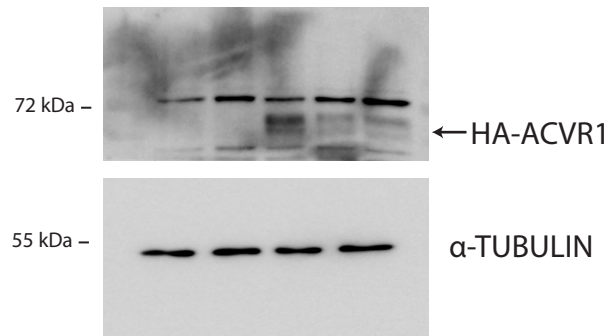

C

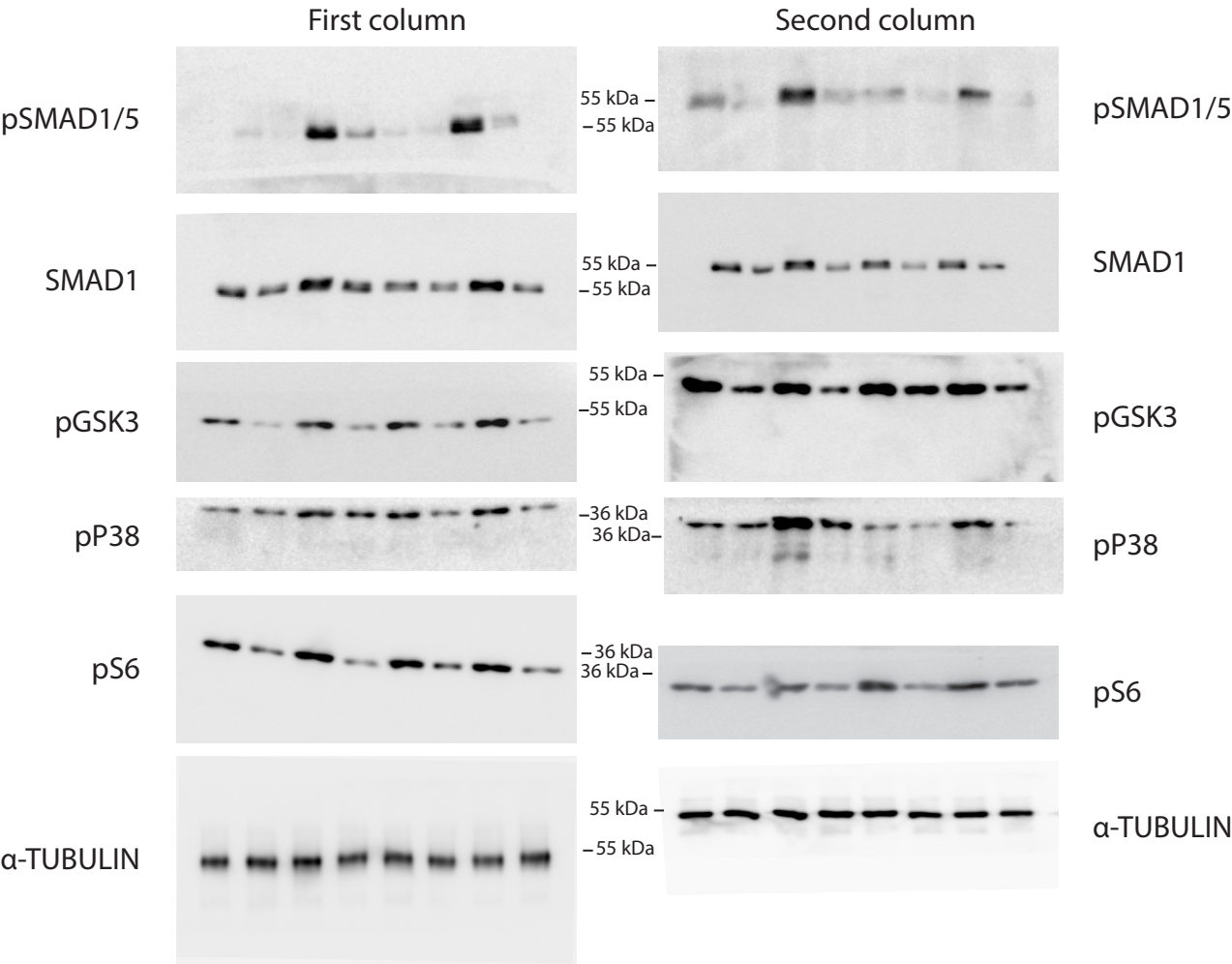

Supplement: Supplementary file 8 — Source Data for Figure 1 [file EMMM-11-e10567-s006.pdf]
